# School Environment and Sanitation in Rural India

**DOI:** 10.4103/0974-777X.62882

**Published:** 2010

**Authors:** J P Majra, A Gur

**Affiliations:** *Department of Community Medicine, K.S. Hegde Medical Academy, Mangalore-575 018, India*; 1 *Department of Periodontics, A. B. Shetty Memorial Institute of Dental Sciences, Mangalore, India*

**Keywords:** Hygiene behavior, India, School environment, School children, Sanitation

## Abstract

**Context:**

A school child educated about the benefits of sanitation and good hygiene behavior is a conduit for carrying those messages far beyond the school walls, bringing lasting improvement to community hygienic practices.

**Aims:**

To study the status of school environment and sanitation in rural India.

**Settings and Design:**

Government schools in rural Karnataka, cross sectional study.

**Materials and Methods:**

Twenty schools were randomly selected for the study. Informed consent was taken from the Heads of the schools. A pre tested close ended questionnaire was used to get the information. The minimum standards for sanitation of the school and its environment in India were used as the guiding principles to evaluate the appropriateness/ adequacy of the various attributes.

**Statistical analysis used:**

Percentages and proportions.

**Results:**

Out of 20 schools selected, one fourth of the schools were located/ sited at inappropriate places. Only half of the schools had appropriate/ adequate structure. Eighteen (90%) of the schools were overcrowded. Ventilation and day light was adequate for 12(60%) and 14(70%) of the schools respectively. Cleanliness of school compound/classrooms was adequate in 80% of the schools. There were no separate rooms for serving the midday meals in any of the schools under study. Eighteen (90%) of the schools were having drinking water points. Liquid and solid waste disposal was insanitary in six (30%) and eight (40%) of the schools respectively. Only half of the schools had adequate latrines for boys and 60% for girls. Only two (10%) of the schools had adequate hand washing points with soap.

**Conclusions:**

Environment and sanitation facilities at many of the schools are not fully satisfactory.

## INTRODUCTION

In light of the strong interaction between sanitation and health, education, malnutrition and poverty, and of insufficient progress towards improving sanitation, 2008 was declared the International Year of Sanitation. India is one of the largest countries of the world with diverse population both in geographical and cultural terms. Given this, it has been a challenge to universally provide safe drinking water and sanitation facilities in India. As per NFHS-3, 88% of the total population of the country had access to improved source of drinking water and 45% had latrines within their households. This was even less in rural areas i.e. 85% and 26%, and out of this, only 18% households have latrines with water closet.[1] Furthermore, inadequate use of water and sanitation facilities and poor hygiene practices has enhanced the severity of such challenges. Open defecation remains the predominant norm and poses one of the biggest threats to the health of the people in India. Estimates suggest that nearly 65% of India's population still defecate in the open. This practice of open defecation is reinforced by traditional behavior patterns and lack of awareness about the health threats posed by it.[2] The best way to break bad practices is to cultivate good practices and childhood is the best time for that as children are receptive to all influences. Therefore school sanitation and hygiene education have been given prominence in the Total Sanitation Campaign, which recognizes the role of children in absorbing and popularizing new ideas and concepts.[3] This program intends to tap their potential as the most persuasive advocates of good sanitation practices in their own schools, households and community. Government of India is committed to scale up School Sanitation and Hygiene Education program by covering all the government rural schools with water, urinal/toilet facilities and promotes health and hygiene activities by the fiscal year 2005-06 with special focus on girl child.[4] Unfortunately the promises of school health and hygiene education programs have not always been fulfilled.[5] Therefore, this study was carried out to know the ground realities as far as school environment and sanitation is concerned.

## MATERIALS AND METHODS

Our study was carried in the rural areas of Mangalore Taluk in Dakashan Kannada District of Karnataka state in India. A list of the government schools in the taluk was obtained. Twenty schools were randomly selected for the purpose of study from the list. Heads of the schools were contacted and were informed about the purpose of the study. They were ensured that a total confidentiality of identity shall be maintained. Their informed consent was taken. A pre tested close ended questionnaire was used to get the information. A group of medical interns were trained to collect the data for the study. Information on parameters such as location, site, structure, classrooms, ventilation and lighting, water supply, eating facilities, latrine, urinals and hand washing facilities in the schools was collected. The minimum standards for sanitation of the school and its environment in India given elsewhere[6] were used as the guiding principles to evaluate the appropriateness/ adequacy of the various attributes related to school sanitation and environment selected for the study. Data thus collected were compiled and analyzed. Percentages and proportions were used as the statistical methods.

## RESULTS

The study was carried in rural areas of Mangalore Taluk in Dakashan Kannada District of Karnataka. A total of 20 randomly selected government schools were studied for their environment and sanitation facilities. Out of these twenty schools, four schools were primary schools, 14 were primary plus upper primary and two schools were from primary to high school level. It was observed [Table 1] that 15 (75%) of the schools were centrally placed with approach roads and at a fair distance from the busy places and roads. These were properly fenced and sited at properly drained high land. One fourth of the schools were located/ sited at inappropriate places. Only half of the schools had appropriate/ adequate structure as per recommendations of the School Health Committee. Eighteen (90%) of the schools had less than 10 square feet per capita space in the classrooms and hence were overcrowded. Ventilation in terms of floor, door/window area was observed to be adequate for 12(60%) of the schools but only eight (40%) schools were having classrooms with cross ventilation. Day light was observed to be inadequate in almost one third (30%) of the schools. Cleanliness of school compound/classrooms was observed to be adequate in 80% of the schools. However, there were no separate rooms to serve the midday meals in any of the schools under study. Either verandas or classrooms were being used for serving midday meals. Most 18(90%) of the schools were having drinking water points which were adequate/appropriate, while drainage of waste water was appropriate in only 70% of the schools. In six (30%) of the schools waste water was seen stagnating and was a risk factor for breeding of mosquitoes. Solid waste including kitchen waste was indiscriminately dumped in eight (40%) of the schools. Latrines were grossly inadequate for boys as well as girls. Only half of the schools were having required number of latrines for boys and 60% were having required number of latrines for girls. None of the schools were having any separate urinals. The students were using the latrines available in the schools for urination too. Hand washing facilities were pitiable in most of the schools only two (10%) of the schools were having adequate hand washing points with soap.

**Table 1:** School environment and sanitation in rural Karnataka

Attribute	Appropriate/adequate %	Inappropriate/inadequate %	Total %
Location and site	15(75)	5(25)	20(100)
Structure	10(50)	10(50)	20(100)
Space	2(10)	18(90)	20(100)
Ventilation	12(60)	8(40)	20(100)
Lighting	14(70)	6(30)	20(100)
Cleanliness of school compound/classrooms	16(80)	4(20)	20(100)
Eating facilities	0(0)	20(100)	20(100)
Water point/s	18(90)	2(10)	20(100)
Drainage of waste water	14(70)	6(30)	20(100)
Solid waste disposal	12(60)	8(40)	20(100)
Latrines for boys	10(50)	10(50)	20(100)
Latrines for girls	12(60)	8(40)	20(100)
Urinals for boys	0(0)	20(100)	20(100)
Urinals for girls	0(0)	20(100)	20(100)
Hand washing facilities	4(20)	16(80)	20(100)

All figures in parenthesis are in percentage

## DISCUSSION

The National Health Policy 2002 acknowledges that despite the impressive public health gains, morbidity and mortality levels in the country are still unacceptably high and that these unsatisfactory health indices are, in turn, an indication of the limited success of the public health system in meeting the preventive and curative requirements of the general population. The common water-borne infections – Gastroenteritis, Cholera, and some forms of Hepatitis – continue to contribute to a high level of morbidity in the population, even though the mortality rate may have been somewhat moderated.[7] Morbidity and mortality due to waterborne diseases have not declined commensurate with increase in availability of potable water supply, largely owing to the fact that quality of water is not maintained at consumer point and that safe water may become contaminated during storage due to poor handling practices and poor personal hygiene.[7] Therefore, the availability of potable water alone may not result in significant decline in water-borne diseases, especially diarrhea, unless the quality of water is also ensured at consumer point, and significant improvements in hygiene behavior take place. Experience shows that involving children as agents of change helped transform the mindset of communities and facilitated the widespread adoption of hygienic practices.[8] School is important for cognitive, creative and social development of children. A school child educated to the benefits of sanitation and good hygiene behavior is a conduit for carrying those messages far beyond the school walls, bringing lasting improvement not only to his or her health and wellbeing, but also to that of the family and the wider community.[9] Perhaps the most important lesson from past experience is that School Sanitation and Hygiene Education is an ‘approach to life’ rather than an academic subject that can be taught with a focus on theory and written examinations. With that in mind classroom teaching has to go hand in hand with practice and that in turn demands that schools have adequate, clean and well maintained water and sanitation facilities.[9] Those are the foundations for providing schools with clean drinking water and well-designed and maintained sanitation facilities; a healthy school environment and a platform for competent teaching to implant the hygiene habits that can bring lasting benefits to entire communities. This understanding is very much a part of the policy of Government of India and school sanitation programs are being given high priority in the successive Five Year Plans. Our study shows that a lot have been done and much more is required to be done on the issue of school environment and sanitation. The number of rural schools of all categories has gone to more than one million out of which 45.9% are without toilets and only 17.3% are without water supply as projected by Ministry of Human Resource Development, Government of India.[10] The challenges, therefore, are enormous and substantial.

## CONCLUSION

The present environment and sanitation facilities at many of the schools are not fully satisfactory. Therefore, in order to tap the potential of the school children as the most persuasive advocates of good sanitation practices in the community, it required to provide them with a healthy school environment.
